# Correction: Lack of ANKMY2 suppresses kidney cystogenesis in embryonic- and adult-onset polycystic kidney disease

**DOI:** 10.1371/journal.pgen.1012049

**Published:** 2026-02-17

**Authors:** The PLOS Genetics Staff

S1 Data was uploaded incorrectly. Please see the correct S1 Data below.

The publisher apologizes for the error.

## Supporting information

S1 Data Numerical data in figures and supplemental figures. All numerical data in figures and supplemental figures are provided in this spreadsheet.
(XLSX)
