# Supplementary material for: Lack of ANKMY2 suppresses kidney cystogenesis in embryonic- and adult-onset polycystic kidney disease
Source: PLoS Genet. 2025 Dec 31;21(12):e1012008. doi: 10.1371/journal.pgen.1012008 (PMC12774363; doi:10.1371/journal.pgen.1012008)
Supplement: S1 Data — All numerical data in figures and supplemental figures are provided in this spreadsheet. (DOCX) [file pgen.1012008.s008.docx]

Cover blurb

ANKMY2 regulates cystic burden in embryonic- and adult-onset polycystic kidney disease in mouse models. Immunostaining of the kidney section of a *HoxB7-Cre*; *Pkd1^f/f;^ Ankmy2^f/f^* mouse harvested at postnatal day 3 shown depicts partially reduced cysts in Aquaporin 2 marked collecting ducts (magenta) compared to *HoxB7-Cre*; *Pkd1^f/f^* (not shown). Proximal tubules (marked by the labeled Lotus Tetragonolobus Lectin in green) are not targeted in this embryonic-onset polycystic kidney disease mouse model. Counterstaining of nuclei using DAPI (blue). See *Hwang* et al.

Image credit: Kyungsuk Choi and Sun-Hee Hwang.
