# Supplementary material for: Lack of ANKMY2 suppresses kidney cystogenesis in embryonic- and adult-onset polycystic kidney disease
Source: PLoS Genet. 2025 Dec 31;21(12):e1012008. doi: 10.1371/journal.pgen.1012008 (PMC12774363; doi:10.1371/journal.pgen.1012008)
Supplement: Attachment — Submitted filename: Responses to ReviewerComments_Hwang.pdf [file pgen.1012008.s010.pdf]

We wholeheartedly thank the reviewers for their comments and constructive critique. We appreciate the positive response from the editors and reviewers (for e. g., Reviewer 1: "*novel and complementary to other results in the field*"; Reviewer 2: "*interesting study*"). We believe that we have now addressed most of the remaining concerns to the best of our capacity and practicability and revised the manuscript according to the suggestions. These changes have resulted in the following changes:

- Revised Figure S5 adding new data on cilia lengths in *Ankmy2* ko cultured cells.
- Revised Figure S5 adding new data on role of ANKMY2 in trafficking of endogenous ADCY3 in human ADPKD cyst-lining cells and normal human kidney primary cells.
- Revised title to "Lack of ANKMY2…" from "Lack of adenylyl cyclase targeting to primary cilia…" based on the reviewers' suggestions regarding data interpretations.
- Textual edits based on the reviewers' suggestions regarding data interpretations.
- Adding a section in Discussion on current limitations in studying ciliary adenylyl cyclase/cAMP signaling in ADPKD models.
- Additional data for reviewers on pCREB immunostaining in cilia in PKD mouse models.
- Additional data on ciliary length in pre-cystic *Ankmy2* conditional knockout kidney tubules at different stages and in both sexes (Figure S6, S7).
- Reordering of Figures 6 and 7.
- Extended data showing raw values of graphs in Figures.
- Updated authorship based on new data using NHK and human ADPKD primary renal epithelial cells.

Revised text in the manuscript is marked.

Reviewer #1: The manuscript by Hwang et al. entitled „Lack of adenylyl cyclase targeting to 5 of 8 primary cilia suppresses kidney cystogenesis in embryonic- and adult-onset polycystic kidney disease" investigates the role of ciliary AC/cAMP signaling controlled by ANKMY2 in cystogenesis. The authors generated kidney-specific Ankmy2/Pkd1 knockout mice, demonstrating that loss of ANKMY2 suppressed early postnatal cystogenesis and prolonged survival. Mechanistically, the authors attribute this to inhibition of the ciliary length increase, which is generally observed in ADPKD models. The authors conclude that ciliary ACs promote cystogenesis and that these mechanisms are distinct from the cAMP-dependent mechanisms in the cytosol. The results are novel and complementary to other results in the field, demonstrating that ciliary cAMP is the main driver of cystogenesis.

Thanks for the comments.

However, the conceptual advance in terms of mechanistic insights needs to be further improved. Thus, the following points need to be addressed:
1. Figure 2/5: The authors use pCREB as a read-out for total cAMP production in the cells, which is only a proxy. This should be analyzed by sorting epithelial cells and determine total cAMP levels using ELISA, as shown in Fig. 6. The experiment in Figure 6 needs to be done using epithelial cells only if they have been performed using whole tissue. There are easy ways to enrich epithelial cells using MACS (Epcam).

Thanks for the suggestions. The cAMP assays (Enzo Direct cAMP ELISA kit) we perform are routinely performed in kidneys by multiple labs [1-3] and uses a highly sensitive colorimetric ELISA assay (0.006 to 0.027 pmol/ml detection limit). We appreciate the reviewer's comment regarding epithelial enrichment of postnatal or 5-month-old kidneys but please note that such preparations have not been routinely performed and will require *freshly isolated* kidneys from multiple genotypes at different ages (up to 5-month-old animals). In addition, please note that epithelial

enrichment will not resolve the tubule specific differences in cAMP levels. For example, as reported before [4], and also pointed by Reviewer 2, pCREB increase in nucleus does not mark the proximal tubules of adult cystic kidney [4], suggesting heterogeneity in cAMP response between different segments of the tubules. Such heterogeneity in pCREB is also seen in embryonic and early postnatal cystogenesis [5]. Therefore, we respectfully contend that nephron-segment specific epithelial enrichment for measuring total cAMP levels is beyond the scope of our current studies.

2. In addition, there is also literature demonstrating that CREB shuttles through the cilium, where it gets activated by ciliary cAMP/PKA, evoking a gene expression program that is distinct from the cytoplasmic cAMP-dependent program (Hansen et al., 2022). As the authors are analyzing the action of ACs in the cilium, the subcellular localization of pCREB, including the cilium, would need to be analyzed.

Thanks for the suggestion. As mentioned by the reviewer, pCREB have been shown to localize to ciliary tips of photo-activated cultured cells expressing ciliary targeted bPAC [6], and CREB1 has been recently shown to localize to ciliary base of cultured cells[7]. However, as far as we know, pCREB immunofluorescence in kidney sections to measure levels in kidney cilia using *in vivo* models have not been performed. Immunostaining for ciliary tip localized proteins in tissue sections is non-trivial, and even for GLI2 (in Hedgehog pathway) can be challenging in mouse embryonic sections. However, as suggested by the reviewer, we performed immunostaining of P15 and the 5-month-old cryo-protected and cryo-sectioned kidney samples for pCREB, particularly in cystic kidneys with high pCREB levels. Although we detected pCREB localization in nucleus, we were unable to see cilia localization despite multiple attempts in either ciliary tips or ciliary base **(Fig. R1**). There could be multiple reasons for not detecting pCREB in cilia of PKD kidneys: first, the pCREB ciliary levels are too low to detect in PKD kidney epithelia [6], given that previous experiments were using optogenetics in cell lines expressing bacterial photoactivable adenylyl cyclase (bPAC) and second and most likely reason, the current reagents are not sufficient for *in vivo* detection.

3. Figure 2/5: Another major point that is lacking is that no direct measurement for the change of cAMP/PKA signaling in the cilium have been performed. This can be done in isolated cells using biosensors for cAMP and PKA. As biosensor imaging might be difficult in primary cells, the authors should perform those measurements in the generated IMCD-3 cells lacking Ankmy2. These outcome of these experiments could also be correlated with cilia length.

Thanks for the suggestion. Based on published literature of PC1-PC2 functionality as a nonselective cation channel in Ca^{2+} entry that inhibits AC5/6 [8], and recent unpublished results from the Tsiokas lab—following up on previous results on WNT mediated activation of PC1 [9]—demonstrating direct Gαi coupling, PC1/2 activation is expected to reduce cAMP levels in cilia. However, measuring cAMP levels in cilium in wild-type condition will require elucidation of mechanisms for activation of PC1-PC2. Furthermore, measuring *basal* cAMP levels in cilia might not resolve the lack of reduction in cAMP from PC1/2 loss, as the absence of cAMP inhibitory mechanism might not produce a detectable elevation in steady-state cAMP levels. Rather, accurately quantifying cAMP levels in cilium in wild-type condition will require elucidation of mechanisms for activation of PC1-PC2.

We would also like to note that the complexity of ciliary cAMP system and the distinctiveness from the high firing Ca^{2+} signaling is apparent from the basal suppression pathway in vertebrate HH signaling [10]. For example, in the Hedgehog pathway, lack of GPR161—a constitutively active Gαs coupled GPCR—causes increased levels of the transcription factor GLI2 in cilia; however, such mechanisms are mediated from lack of direct PKA regulatory subunit anchoring (AKAP) function of GPR161 distinct from direct Gαs coupling mediated cAMP signaling

[11]. In contrast, high Ca^{2+} signaling in crown cilia in the vertebrate node occurs in bursts of activity from regulated displacement and specific flow-mediated dynamics in a PC2-dependent manner [12,13]. In the absence of known mechanisms regulating activation of PC1-PC2 in kidney epithelial cilia, we respectfully argue that performing such measurements in cilia are beyond the scope of the current manuscript.

Based on the reviewer's comment on cilia lengths, we measured cilia lengths in *Ankmy2* ko IMCD3 cells and find no significant differences with controls. These data are now added in Fig. S5D and are consistent with our previous studies in NIH 3T3 cells [14]. We previously also showed that there were no differences in cilia lengths between control and *Ankmy2* conditional knockout (cko) cilia in proximal tubules and collecting ducts (**Fig. 6B**). Basal cAMP levels in cilia are likely to be equilibrated with cytoplasm unless upon acute stimuli such as GPCR-mediated activation by agonists, or photoactivation of cilia localized bPAC; therefore, although ciliary ACs are not trafficked in *Ankmy2* ko cells, basal levels of ciliary cAMP are not sufficiently reduced to induce shortening of cilia at least in kidney epithelial cells and fibroblasts.

4. Information about the statistical tests is missing in some of the legends 5. All graphs should be presented as mean + S.D. and individual values.

Thanks for the suggestions and sorry for any unintentional oversight. We have added all relevant statistical methods in legends of Fig. 5 in addition to methods. All data are shown as mean ± SD as mentioned in legends. All graphs show individual values and extended data show raw values.

Reviewer #2:
This is an interesting study, where it is suggested that ciliary ADCY5/6-mediated cAMP signaling is downstream of Pkd1-induced ADPKD. The study includes both mouse and cell culture work and if revised, it will provide important mechanistic refinements about the role of the cAMP pathway in ADPKD. However, there are conceptual gaps that need to be addressed. Deletion of PC1 or PC2 leads to an increase in total cellular cAMP levels, as is demonstrated here and in many other publications. This means that PC1 and PC2 function not only in cilia but also in several other places to cause a cellular increase in cAMP upon their deletions. Thus, their contribution to the regulation of cAMP is way more global (at the cellular level) than cAMP in the cilium, which makes sense as they are not there to just prevent cystogenesis. Whether the ciliary cAMP pool is the relevant pool to prevent cystogenesis, however, is an important question and the subject of the current study. This is viewed as a strength of the manuscript. A major general weakness is that conclusions are drawn mostly based on conjecture and not direct evidence. For example, there is no direct evidence that deletion of Ankmy2 leads to the specific depletion of ADCY5/6 in cilia in tissues, no demonstration that there is abnormal cAMP accumulation in Pkd1-null cilia, which is reduced by the depletion of Ankmy2 (even in cell culture), lack of consideration of effects of Ankmy2 on other ADCYs such as ADCY3, and lack of mechanisms of how intraciliary cAMP causes ciliary elongation. While it is not expected that all these issues be addressed in a single manuscript, some key points (see below in specific should be addressed to strengthen the conclusions. The ameliorating phenotypes of Adcy5 or Adcy6 in double mutant mice are extremely weak (PMC5794572, PMC3904559), so it is difficult to see how their partial depletion from cilia can have strong rescuing effects (Figure 3B), especially when deletion of Pkd1 causes a global (cellular) increase in cAMP. Finally, previous work has shown that there is an upregulation of cellular pCREB in collecting ducts, but not in proximal tubules, despite the presence of proximal tubule cysts (PMC3758452). Yet the authors show rescue in late models in both proximal tubules and collecting ducts. It is possible that if the ciliary cAMP pool is relevant to cystogenesis and loss of Pkd1 induces an increase in just the ciliary levels of cAMP in proximal tubules, these cAMP changes may be difficult to capture by looking at total cAMP levels. However, this needs to be

shown. In this regard, accumulation of pCREB in the cilia of both kidney segments may be more telling than total cellular pCREB levels.

Thanks for the comments and suggestions.

As the reviewer mentioned, the ameliorating phenotypes of *Adcy5* or *Adcy6* loss in ADPKD mouse models are partial [15,16]. The strength of ANKMY2, however, lies in being able to target trafficking on multiple adenylyl cyclases that localize to cilia including ADCY3/5/6 [14]. In fact, because of the redundancy of adenylyl cyclases and variability of mechanisms regulating adenylyl cyclases [8], ANKMY2 provides a unique tool to regulate trafficking of adenylyl cyclases targeted to cilia [14]. We previously showed that lack of *Ankmy2* results in complete derepression of hedgehog signaling in mouse embryos in a fully-cilia dependent manner, showing levels of high hedgehog signaling comparable to lack of the hedgehog receptor PTCH1 [14,17] or loss of all PKA subunits [18]. In contrast, overexpression of *ADCY5/6* in the developing chicken neural tube only partially represses the hedgehog pathway [19]; whereas, individual knockdown/knockouts of adenylyl cyclases are only partially penetrant in increased hedgehog signaling phenotypes, and are not embryonic lethal [19,20].

Individual issues raised above are discussed below with respect to specific points.

Specific points
1) Demonstration that depletion of one or more of the ADCYs in cilia in kidney tissues in Ankmy2-null kidneys will be needed. It is understandable that existing antibodies for ADCY5/6 are not reliable but given the lack of clear understanding of how exactly ANKMY2 affects the trafficking of these ADCYs in the cilia, it is an essential point. In this regard, how about ADCY3? Antibodies for this isoform should work on cilia. Whether there is Ca2+ dependance (upon PC1/2 deletion) or not of the cAMP regulation in the cilium is a separate question and if not, there could be alternative explanations for this. Any surrogate assay based on the purported cellular function of ANKMY2 affecting ADCY maturation would suffice. Anything substantial to increase confidence that the presence/function of these ADCYs is reduced specifically in the cilium in Ankmy2-null kidneys would suffice.

Thanks for the comments. Regarding "any surrogate assay based on the purported cellular function of ANKMY2 affecting ADCY maturation", we have already showed in a previous manuscript [14] that the maturation of ADCY3/5/6 are affected in NIH 3T3 cells [14], and that of ADCY5/6 are affected in IMCD3 cells (current manuscript), such that complex glycosylated forms are reduced in both cell lines with or without corresponding increase in core glycosylated forms. The differential effects on the core glycosylated forms in between the cell lines is likely from cell-specific differences in the ERAD pathway in degradation of partially folded proteins in ER [21]. Thus, we have already demonstrated that ANKMY2 prevents maturation of multiple adenylyl cyclases.

Regarding the reviewer's comments on "Anything substantial to increase confidence that the presence/function of these adenylyl cyclases is reduced specifically in the cilium in Ankmy2-null kidneys", we think that the cilia length increases in pre-cystic kidneys in an ANKMY2-dependent manner—that too at a stage when kidney cAMP levels are not increased—suggests that such length increase is likely from cumulative ciliary cAMP changes in ADPKD models. While, understandably, cilia length can increase from other factors, the ANKMY2 dependence strongly argues for ciliary cAMP changes in the absence of total cAMP increase. Please also see detailed response to the next comment.

In addition, and also mentioned in response to Reviewer 1, we diligently tested for ADCY3,5,6 levels in kidney cilia using different fixation conditions and multiple antibodies. However, we were unable to determine these adenylyl cyclases in kidney. Please note that when

we compare expression levels of ADCY3 in mouse mid-gestational embryos versus adult mouse kidney, we see highly significant expression in embryos (up to 2000-fold for ADCY3 compared to ADCY5/6 (please see Fig. S7B in reference [14]), where we were able to detect ADCY3 in the embryonic cilia by immunofluorescence [14]. Furthermore, we also noted reduction in ciliary levels of ADCY3 in *Ankmy2* knockout embryos in the neural tube and mesenchyme even before somitogenesis (E7.75-E8.5) [14]. ADCY3 protein and transcript levels are also lower than ADCY6 and ADCY5 in the kidney tubule as shown by comparative proteomics by the Knepper lab [22] and multi-omics data from Humpherys lab **(Fig. R2**) [23]. Therefore, we think that *in vivo* ADCY3 levels in mouse kidney epithelia are likely below detection limits by immunofluorescence using currently available reagents and not likely because of technical issues from our side.

Finally, based on the reviewer's comments, we further tested normal human kidney (NHK) and human ADPKD cyst lining primary cells. Previous studies from the Wallace lab have suggested a role of ADCY3 in determining cAMP levels in human ADPKD cells [24]. We detected endogenous ADCY3 localization in both NHK and ADPKD cells and further evaluated whether its ciliary localization depends on ANKMY2 using RNAi. We found that efficient knockdown of *ANKMY2* significantly reduced ciliary levels of ADCY3 in both NHK and ADPKD cells compared to control siRNA-treated cells (**Fig. S5D-F**). Overall, our results show that ANKMY2 directs ciliary localization of ADCY5, ADCY6 and ADCY3 in renal epithelial cells and is a core biological mechanism in these cells (as we showed previously for neuroepithelial cells [14]). Thus, ANKMY2 operates the same way in healthy cells as it does in diseased cells from PKD patients. Overall, as *ANKMY2* knockdown similarly reduces ciliary ADCY3 levels in both NHK cells and ADPKD cyst-lining cells, targeting ANKMY2 can be highly significant for translational relevance. These results confirm that the ANKMY2 and the adenylyl cyclase trafficking pathway is conserved in humans, making ANKMY2 a more compelling therapeutic target in ADPKD in reducing cystic burden.

2) Demonstration that there is accumulation of pCREB in the cilium or anything else that would reflect increased cAMP in the cilium in Pkd1-null cells that is reversed in double mutant mice would be needed. Total pCREB level is in disagreement with effects on proximal tubules and ciliary length is not an established readout of increased ciliary cAMP. Experiments in proximal tubules may be more relevant than IMCD cells in this case and could help the authors make their case stronger.

Thanks for the suggestions. We fully agree with the reviewers point that proximal tubule vs collecting duct regulated mechanisms for cAMP mediated cystogenesis could be distinct. The heterogeneity of the cAMP response in between different tubule segments in ADPKD, as eluted to by the reviewer, is shown by distinct nuclear pCREB distributions [4]. Furthermore, total cAMP levels in kidneys that we found to be correlated with severity of cystogenesis as reported earlier [1,2] has limitation in not providing nephron segment-specific read outs. We think that the cilia length increases in pre-cystic kidneys—at a stage when kidney cAMP levels are not increased—in an ANKMY2-dependent manner suggests that such length increase is likely from cumulative ciliary cAMP changes in the adult onset ADPKD models. While, understandably, cilia length can increase from forskolin-induced cellular cAMP changes [25], the ANKMY2 dependence on cilia length increase in the adult onset ADPKD models starting from pre-cystic stages, strongly argues for ciliary cAMP changes in the absence of total cAMP increase. Role of cAMP in cilia length regulation is well documented [25], including optogenetic activation of a cilium targeted bacterial photoactivable adenylyl cyclase (bPAC) that elevates ciliary cAMP levels and induces ciliary lengthening within one hour [6,26].

To further solidify the cilia length regulation results in the context of pre-cystic dilatation stage kidneys, we have now extended these results beyond the 10-week time points in the pre-cystic

kidneys at 8 weeks and 14 weeks in both male and female mice. We find that most tubules are dilatated, and both collecting duct and proximal tubule cilia lengths are increased in male mice at 14 weeks, unlike only proximal tubules at 10 weeks (**Fig. S6, S7**). Similarly, proximal tubule cilia lengths are increased more robustly in female mice at 14 weeks (**Fig. S6, S7**). These results suggest that cilia length increase occurs in both proximal tubules and collecting ducts at pre-cystic stages. However, cilia lengths were not increased at 8 weeks of adult-onset PKD. Combined with the results that such cilia length increase is ANKMY2-dependent at both pre-cystic stages (without overt cAMP changes) and cystic kidneys (with high cAMP levels), and that high cAMP levels in cysts do not cause cilia length increase in absence of ANKMY2, we suggest that ANKMY2 regulation is crucial in the cilia length regulation starting from the pre-cystic stage.

3) What is interpretation that the rescuing effect is lost at P15 (Figure 2)? The deterioration of the phenotype from P3 to P15 argues for a dual effect on Ankmy2/cAMP on cystogenesis.

Thanks for the comment. We think that the deterioration of the rescue of the cystic phenotype from P3 to P15 argues for a reduction of the restorative effects from ANKMY2 loss as cystogenesis progresses. At later time points, although overall survival is extended—likely due to a delayed decline in kidney function—the increasing cystic burden eventually predominates. Through well-established pathways involving disrupted intracellular Ca^{2+} homeostasis, adenylyl cyclase activation, and vasopressin signaling[27], this burden elevates total cellular cAMP and nuclear pCREB levels, ultimately overriding the early inhibitory effects. Cellular cAMP regulates cystogenesis by well documented mechanisms such as fluid secretion [28] through activation of the CFTR chloride channel [29,30] and cell proliferation through activation of the B-Raf/MEK/ERK pathway [31].

4) Is ANKMY2 expressed in Hox7+ and Pax8+ cells?

Ankmy2 is expressed in low levels throughout mouse embryos [14] and adult mouse kidney tubule segments analyzed using longitudinal single cell transcriptomic datasets of control and *Pax8*^{rtTA}; *TetO-Cre*; *Pkd1*$^{f/f}$ kidneys at 9 weeks (P66), 14 weeks (P100) and 4.5 months (P130) [23] (almost similar to our selected time points for the current study) (**Fig. R3**). We also compared mouse kidney transcript levels between genotypes in Fig. S1 from early postnatal kidneys and Fig. S3 from adult 5-month-old kidneys and detect *Ankmy2* transcripts in controls that are reduced upon conditional knockout of *Ankmy2.*

5) Sexual dimorphism is suggested as a possible explanation for the sex differences, but this is not tested.

Thanks for the comment. We compared cystic phenotypes in proximal vs collecting ducts in adult control vs PKD kidneys. We find a rescue of cystic phenotype in both proximal tubule and collecting ducts between *Pkd1* cko va *Pkd1;Ankmy2* dko and find a selective rescue, more pronounced in proximal tubule in males compared to females (Fig. 3F, Fig. S4C). We interpret these data as sex-specific effects on tubule to cyst formation in ADPKD and selective rescue from lack of ANKMY2. In addition, in *Pax8*^{rtTA}; *TetO-Cre*; *Pkd1*$^{f/f}$ females, ciliary lengthening in the proximal tubule at the tubule dilatation stage was less robust compared to males. The slower initial ciliary lengthening during the tubule dilatation stages in females suggest that the ciliary component of adenylyl cyclase signaling derepressed from polycystin loss is temporally delayed and/or overlaps with downstream cytoplasmic pathways precluding efficient ANKMY2-mediated suppression of cystic burden. We removed the term “sexual dimorphism” as we are discussing about sex-specificity in phenotypes. The lack of effect in female in contrast to male mice suggested sex-specificity in effects of ANKMY2 in the PKD model most likely from androgen

receptor mediated control of sexually dimorphic gene expression in tubule segments [32,33]. The sex-specificity aligns with the well-documented sex-related differences in both human ADPKD patients and rodent models, which exhibit more severe cystic disease in males than in females [34,35].

6) Experiments in cell culture are difficult to interpret (Figure 5 and S5). Levels of glycosylation of ADCY5/6 are reduced in ANKMY2 KO cells, but how does this affect ciliary expression versus plasma membrane expression? The data are more consistent with a global effect of ANKMY2 on maturation and processing of ADCY5/6 towards both the plasma and cilia membrane, unless the authors suggest that ANKMY2 promotes/facilitates the lateral movement of these ADCYs from the plasma membrane to the cilium via the transition zone or similar structures that control ciliary entry of transmembrane proteins. If so, this needs to be shown. The conclusion in lines 298-301, p. 12 is unsupported. How about cilia length in these cells?

Thanks for the suggestions. As the reviewer rightly points out, parsing out the effects of ciliary levels of ADCYs from plasma membrane pools would require determining factors that selectively mediate lateral movement into cilia. ADCY3 has been shown to be a bona fide IFT-A ciliary cargo in embryonic contexts [36]. However, interpretation of IFT-A or the adapter TULP3 in regulation of ADCYs are complicated by role of IFT-A in retrograde IFT and of TULP3/IFT-A in regulating transport of multiple types of ciliary cargoes. Thus, dissecting specific role of ciliary compartmentalization of multiple adenylyl cyclases would require mutants that prevent ciliary localization while still retaining cellular pools and without disrupting cilia, beyond the scope of the current study. When we write in lines 298-301 that “our data from renal epithelial cell lines combined with nephron segment-specific proteomic profiles [22] suggest that conditional knockouts of *Ankmy2* prevented ciliary localization of multiple adenylyl cyclases in the kidney tubules.”, we were generalizing the effects from our cultured cells data and from other systems.

We previously showed that there was no difference in cilia lengths between control and *Ankmy2* cko cilia in proximal tubules and collecting ducts (**Fig. 6B**). Furthermore, we have now measured cilia lengths between IMCD3 control parental and *Ankmy2* ko cell lines and find no differences in cilia length (Revised **Fig. S5D**), results that are consistent with our previous studies in NIH 3T3 cells [14]. Basal cAMP levels in cilia are likely to be equilibrated with cytoplasm unless there are activation stimuli such as GPCR-mediated activation by agonists, or photoactivation of cilia localized bPAC; therefore, although ciliary ACs are not trafficked in *Ankmy2* ko cells, basal levels of ciliary cAMP are not sufficiently reduced to induce shortening of cilia in these cells.

We showed that stably overexpressed ADCY5/6 are localized to the plasma membrane and ANKMY2 also regulates plasma membrane pools. However, given the effect on ciliary length control in adult-onset ADPKD in an ANKMY2-dependent manner, and ciliary length control being mediated by factors that localize in the cilia-centrosomal complex [37,38], the most parsimonious model would be that the effect on ciliary/peri-ciliary adenylyl cyclase signaling by ANKMY2 is the major contributing factor in regulating cystic burden in ADPKD (**Fig. 8**). Similarly, high hedgehog signaling from pathway derepression in the *Ankmy2* ko mouse neural tube was fully cilia-dependent [14]. In the kidney tubules, lack of ciliary/peri-ciliary adenylyl cyclase signaling from ANKMY2 loss suppresses cyst initiation but could be less effective at later stages when cellular cAMP levels rise during cyst progression.

7) Figure 7E. cAMP levels represent total levels. These data are confusing. There is a positive correlation between cAMP levels and cystic index in single and double mutants. How can it possible, if Ankmy2 only affects the ciliary pool that should generate undetectable levels of cAMP?

I’d expect positive correlation in single ko male mice, but no correlation of total cAMP and cystic index in male dko mice.

We are sorry that these data were not clear enough to the reviewer. We would like to reiterate here that total kidney cAMP levels are reflective of cystic index irrespective of genotype. Such correlations between cystic index and cAMP levels have been reported before [1,2]. However, in this paper, we note that the ciliary lengths in *Pkd1* mutant animals start to increase in pre-cystic dilatation stage kidney tubules, quicker in males than females, but do not show any increase from pre-cystic stages to cystic kidneys in *Pkd1; Ankmy2* double mutants. Given that previous work has shown that ciliary cAMP results in ciliary length increase [6], we interpret these data to suggest that ciliary cAMP results in pre-cystic dilatation stage increase in ciliary length. Elongated cilia could reflect signaling by ciliary and periciliary effectors promoting cystogenesis. Alternatively, elongated cilia could alter cilia-generated signaling by modulating the activity of ciliary and periciliary effectors induced by flow. Identifying ciliary effectors of ciliary length control at early stages could point to ciliary mediators of pre-cystic changes determining cystogenesis.

References

1. Arroyo, J., Escobar-Zarate, D., Wells, H.H., Constans, M.M., Thao, K., Smith, J.M., Sieben, C.J., Martell, M.R., Kline, T.L., Irazabal, M.V., et al. (2021). The genetic background significantly impacts the severity of kidney cystic disease in the Pkd1(RC/RC) mouse model of autosomal dominant polycystic kidney disease. Kidney Int *99*, 1392-1407. 10.1016/j.kint.2021.01.028.

2. Hopp, K., Ward, C.J., Hommerding, C.J., Nasr, S.H., Tuan, H.F., Gainullin, V.G., Rossetti, S., Torres, V.E., and Harris, P.C. (2012). Functional polycystin-1 dosage governs autosomal dominant polycystic kidney disease severity. J Clin Invest *122*, 4257-4273. 10.1172/JCI64313.

3. Choi, Y.H., Suzuki, A., Hajarnis, S., Ma, Z., Chapin, H.C., Caplan, M.J., Pontoglio, M., Somlo, S., and Igarashi, P. (2011). Polycystin-2 and phosphodiesterase 4C are components of a ciliary A-kinase anchoring protein complex that is disrupted in cystic kidney diseases. Proc Natl Acad Sci U S A *108*, 10679-10684. 10.1073/pnas.1016214108.

4. Ma, M., Tian, X., Igarashi, P., Pazour, G.J., and Somlo, S. (2013). Loss of cilia suppresses cyst growth in genetic models of autosomal dominant polycystic kidney disease. Nat Genet *45*, 1004-1012. 10.1038/ng.2715.

5. Puri, P., Schaefer, C.M., Bushnell, D., Taglienti, M.E., Kreidberg, J.A., Yoder, B.K., and Bates, C.M. (2018). Ectopic Phosphorylated Creb Marks Dedifferentiated Proximal Tubules in Cystic Kidney Disease. Am J Pathol *188*, 84-94. 10.1016/j.ajpath.2017.09.015.

6. Hansen, J.N., Kaiser, F., Leyendecker, P., Stuven, B., Krause, J.H., Derakhshandeh, F., Irfan, J., Sroka, T.J., Preval, K.M., Desai, P.B., et al. (2022). A cAMP signalosome in primary cilia drives gene expression and kidney cyst formation. EMBO Rep *23*, e54315. 10.15252/embr.202154315.

7. Hansen, J.N., Sun, H., Kahnert, K., Westenius, E., Johannesson, A., Villegas, C., Le, T., Tzavlaki, K., Winsnes, C., Pohjanen, E., et al. (2025). Intrinsic heterogeneity of primary cilia revealed through spatial proteomics. Cell. 10.1016/j.cell.2025.08.039.

8. Halls, M.L., and Cooper, D.M. (2011). Regulation by Ca2+-signaling pathways of adenylyl cyclases. Cold Spring Harb Perspect Biol *3*, a004143. 10.1101/cshperspect.a004143.

9. Kim, S., Nie, H., Nesin, V., Tran, U., Outeda, P., Bai, C.X., Keeling, J., Maskey, D., Watnick, T., Wessely, O., and Tsiokas, L. (2016). The polycystin complex mediates Wnt/Ca(2+) signalling. Nat Cell Biol *18*, 752-764. 10.1038/ncb3363.

10. Kopinke, D., Norris, A.M., and Mukhopadhyay, S. (2020). Developmental and regenerative paradigms of cilia regulated hedgehog signaling. Semin Cell Dev Biol. 10.1016/j.semcdb.2020.05.029.

11. Hoppe, N., Harrison, S., Hwang, S.H., Chen, Z., Karelina, M., Deshpande, I., Suomivuori, C.M., Palicharla, V.R., Berry, S.P., Tschaikner, P., et al. (2024). GPR161 structure uncovers the redundant role of sterol-regulated ciliary cAMP signaling in the Hedgehog pathway. Nat Struct Mol Biol *31*, 667-677. 10.1038/s41594-024-01223-8.

12. Djenoune, L., Mahamdeh, M., Truong, T.V., Nguyen, C.T., Fraser, S.E., Brueckner, M., Howard, J., and Yuan, S. (2023). Cilia function as calcium-mediated mechanosensors that instruct left-right asymmetry. Science *379*, 71-78. 10.1126/science.abq7317.

13. Katoh, T.A., Omori, T., Mizuno, K., Sai, X., Minegishi, K., Ikawa, Y., Nishimura, H., Itabashi, T., Kajikawa, E., Hiver, S., et al. (2023). Immotile cilia mechanically sense the direction of fluid flow for left-right determination. Science *379*, 66-71. 10.1126/science.abq8148.

14. Somatilaka, B.N., Hwang, S.H., Palicharla, V.R., White, K.A., Badgandi, H., Shelton, J.M., and Mukhopadhyay, S. (2020). Ankmy2 Prevents Smoothened-Independent Hyperactivation of the Hedgehog Pathway via Cilia-Regulated Adenylyl Cyclase Signaling. Dev Cell *54*, 710-726 e718. 10.1016/j.devcel.2020.06.034.

15. Wang, Q., Cobo-Stark, P., Patel, V., Somlo, S., Han, P.L., and Igarashi, P. (2018). Adenylyl cyclase 5 deficiency reduces renal cyclic AMP and cyst growth in an orthologous mouse model of polycystic kidney disease. Kidney Int *93*, 403-415. 10.1016/j.kint.2017.08.005.

16. Rees, S., Kittikulsuth, W., Roos, K., Strait, K.A., Van Hoek, A., and Kohan, D.E. (2014). Adenylyl cyclase 6 deficiency ameliorates polycystic kidney disease. J Am Soc Nephrol *25*, 232-237. 10.1681/ASN.2013010077.

17. Hwang, S.H., White, K.A., Somatilaka, B.N., Wang, B., and Mukhopadhyay, S. (2023). Context-dependent ciliary regulation of hedgehog pathway repression in tissue morphogenesis. PLoS Genet *19*, e1011028. 10.1371/journal.pgen.1011028.

18. Tuson, M., He, M., and Anderson, K.V. (2011). Protein kinase A acts at the basal body of the primary cilium to prevent Gli2 activation and ventralization of the mouse neural tube. Development *138*, 4921-4930. 10.1242/dev.070805.

19. Vuolo, L., Herrera, A., Torroba, B., Menendez, A., and Pons, S. (2015). Ciliary adenylyl cyclases control the Hedgehog pathway. J Cell Sci *128*, 2928-2937. 10.1242/jcs.172635.

20. Wang, Z., Li, V., Chan, G.C., Phan, T., Nudelman, A.S., Xia, Z., and Storm, D.R. (2009). Adult type 3 adenylyl cyclase-deficient mice are obese. PLoS One *4*, e6979. 10.1371/journal.pone.0006979.

21. Tsai, Y.C., and Weissman, A.M. (2010). The Unfolded Protein Response, Degradation from Endoplasmic Reticulum and Cancer. Genes Cancer *1*, 764-778. 10.1177/1947601910383011.

22. Mehta, Y.R., Lewis, S.A., Leo, K.T., Chen, L., Park, E., Raghuram, V., Chou, C.L., Yang, C.R., Kikuchi, H., Khundmiri, S., et al. (2022). "ADPKD-omics": determinants of cyclic AMP levels in renal epithelial cells. Kidney Int *101*, 47-62. 10.1016/j.kint.2021.10.014.

23. Muto, Y., Yoshimura, Y., Wu, H., Chang-Panesso, M., Ledru, N., Woodward, O.M., Outeda, P., Cheng, T., Mahjoub, M.R., Watnick, T.J., and Humphreys, B.D. (2024). Multiomics profiling of mouse polycystic kidney disease progression at a single-cell resolution. Proc Natl Acad Sci U S A *121*, e2410830121. 10.1073/pnas.2410830121.

24. Pinto, C.S., Reif, G.A., Nivens, E., White, C., and Wallace, D.P. (2012). Calmodulin-sensitive adenylyl cyclases mediate AVP-dependent cAMP production and Cl- secretion

by human autosomal dominant polycystic kidney cells. Am J Physiol Renal Physiol *303*, F1412-1424. 10.1152/ajprenal.00692.2011.

25. Besschetnova, T.Y., Kolpakova-Hart, E., Guan, Y., Zhou, J., Olsen, B.R., and Shah, J.V. (2010). Identification of signaling pathways regulating primary cilium length and flow-mediated adaptation. Curr Biol *20*, 182-187. 10.1016/j.cub.2009.11.072.

26. Hansen, J.N., Kaiser, F., Klausen, C., Stuven, B., Chong, R., Bonigk, W., Mick, D.U., Moglich, A., Jurisch-Yaksi, N., Schmidt, F.I., and Wachten, D. (2020). Nanobody-directed targeting of optogenetic tools to study signaling in the primary cilium. Elife *9*. 10.7554/eLife.57907.

27. Wang, X., Ward, C.J., Harris, P.C., and Torres, V.E. (2010). Cyclic nucleotide signaling in polycystic kidney disease. Kidney Int *77*, 129-140. 10.1038/ki.2009.438.

28. Wallace, D.P., Rome, L.A., Sullivan, L.P., and Grantham, J.J. (2001). cAMP-dependent fluid secretion in rat inner medullary collecting ducts. Am J Physiol Renal Physiol *280*, F1019-1029. 10.1152/ajprenal.2001.280.6.F1019.

29. Magenheimer, B.S., St John, P.L., Isom, K.S., Abrahamson, D.R., De Lisle, R.C., Wallace, D.P., Maser, R.L., Grantham, J.J., and Calvet, J.P. (2006). Early embryonic renal tubules of wild-type and polycystic kidney disease kidneys respond to cAMP stimulation with cystic fibrosis transmembrane conductance regulator/Na(+),K(+),2Cl(-) Co-transporter-dependent cystic dilation. J Am Soc Nephrol *17*, 3424-3437. 10.1681/ASN.2006030295.

30. Hanaoka, K., and Guggino, W.B. (2000). cAMP regulates cell proliferation and cyst formation in autosomal polycystic kidney disease cells. J Am Soc Nephrol *11*, 1179-1187. 10.1681/ASN.V1171179.

31. Yamaguchi, T., Pelling, J.C., Ramaswamy, N.T., Eppler, J.W., Wallace, D.P., Nagao, S., Rome, L.A., Sullivan, L.P., and Grantham, J.J. (2000). cAMP stimulates the in vitro proliferation of renal cyst epithelial cells by activating the extracellular signal-regulated kinase pathway. Kidney Int *57*, 1460-1471. 10.1046/j.1523-1755.2000.00991.x.

32. Xiong, L., Liu, J., Han, S.Y., Koppitch, K., Guo, J.J., Rommelfanger, M., Miao, Z., Gao, F., Hallgrimsdottir, I.B., Pachter, L., et al. (2023). Direct androgen receptor control of sexually dimorphic gene expression in the mammalian kidney. Dev Cell *58*, 2338-2358 e2335. 10.1016/j.devcel.2023.08.010.

33. Wu, H., Lai, C.F., Chang-Panesso, M., and Humphreys, B.D. (2020). Proximal Tubule Translational Profiling during Kidney Fibrosis Reveals Proinflammatory and Long Noncoding RNA Expression Patterns with Sexual Dimorphism. J Am Soc Nephrol *31*, 23-38. 10.1681/ASN.2019040337.

34. Cowley, B.D., Jr., Rupp, J.C., Muessel, M.J., and Gattone, V.H., 2nd (1997). Gender and the effect of gonadal hormones on the progression of inherited polycystic kidney disease in rats. Am J Kidney Dis *29*, 265-272. 10.1016/s0272-6386(97)90039-1.

35. Gabow, P.A., Johnson, A.M., Kaehny, W.D., Kimberling, W.J., Lezotte, D.C., Duley, I.T., and Jones, R.H. (1992). Factors affecting the progression of renal disease in autosomal-dominant polycystic kidney disease. Kidney Int *41*, 1311-1319. 10.1038/ki.1992.195.

36. Liem, K.F., Jr., Ashe, A., He, M., Satir, P., Moran, J., Beier, D., Wicking, C., and Anderson, K.V. (2012). The IFT-A complex regulates Shh signaling through cilia structure and membrane protein trafficking. J Cell Biol *197*, 789-800. 10.1083/jcb.201110049.

37. Avasthi, P., and Marshall, W.F. (2012). Stages of ciliogenesis and regulation of ciliary length. Differentiation *83*, S30-42. 10.1016/j.diff.2011.11.015.

38. Keeling, J., Tsiokas, L., and Maskey, D. (2016). Cellular Mechanisms of Ciliary Length Control. Cells *5*. 10.3390/cells5010006.

Reviewer figure legends.

Figure R1. pCREB subcellular localization in P15 and adult mouse kidneys. Kidneys from *Hoxb7-Cre; Pkd1*$^{f/f}$ at P15 and *Pax8*^{rtTA}; *TetO-Cre*; *Pkd1*$^{f/f}$ at 5 months were perfused, fixed, cryosectioned and immunostained against pCREB (9198S; Cell Signaling, 1:500) and acetylated tubulin, and counterstained using DAPI. Although nuclear pCREB was easily seen we were unable to visualize ciliary or periciliary pCREB.

Figure R2. scRNA data on kidney ACs [23]. Data from control and *Pax8*^{rtTA}; *TetO-Cre*; *Pkd1*$^{f/f}$ at postnatal day 66 (P66, 9 weeks), 100 (P100, 14 weeks), or 130 (P130, 4.5 months), with doxcycline treatment from 4-6 weeks. Abbreviations: PTS1/S2/S3, proximal tubule S1/S2/S3 segments; FRPTC_PEC, failed-repair proximal tubular cells and parietal epithelial cells; DTL1/DTL2/ATL, descending thin limb 1/2 and ascending thin limb of Henle's loop; TAL, thick ascending limb of Henle's loop; DCT, distal convoluted tubule; CNT, connecting tu bule; PC1/2, principle cells 1/2; URO, uroepithelial cells; ICA, Type A intercalated cells; ICB, Type B intercalated cells; PODO, podocytes; ENDO, endothelial cells; FIB, fibroblasts; Myel, myeoloid cells; FAT, adipocyte.

Figure R3. *Ankmy2* expression is tubule segments using scRNA analyses [23]. Data as in Figure R2. Abbreviations as in R3.

FIG R1

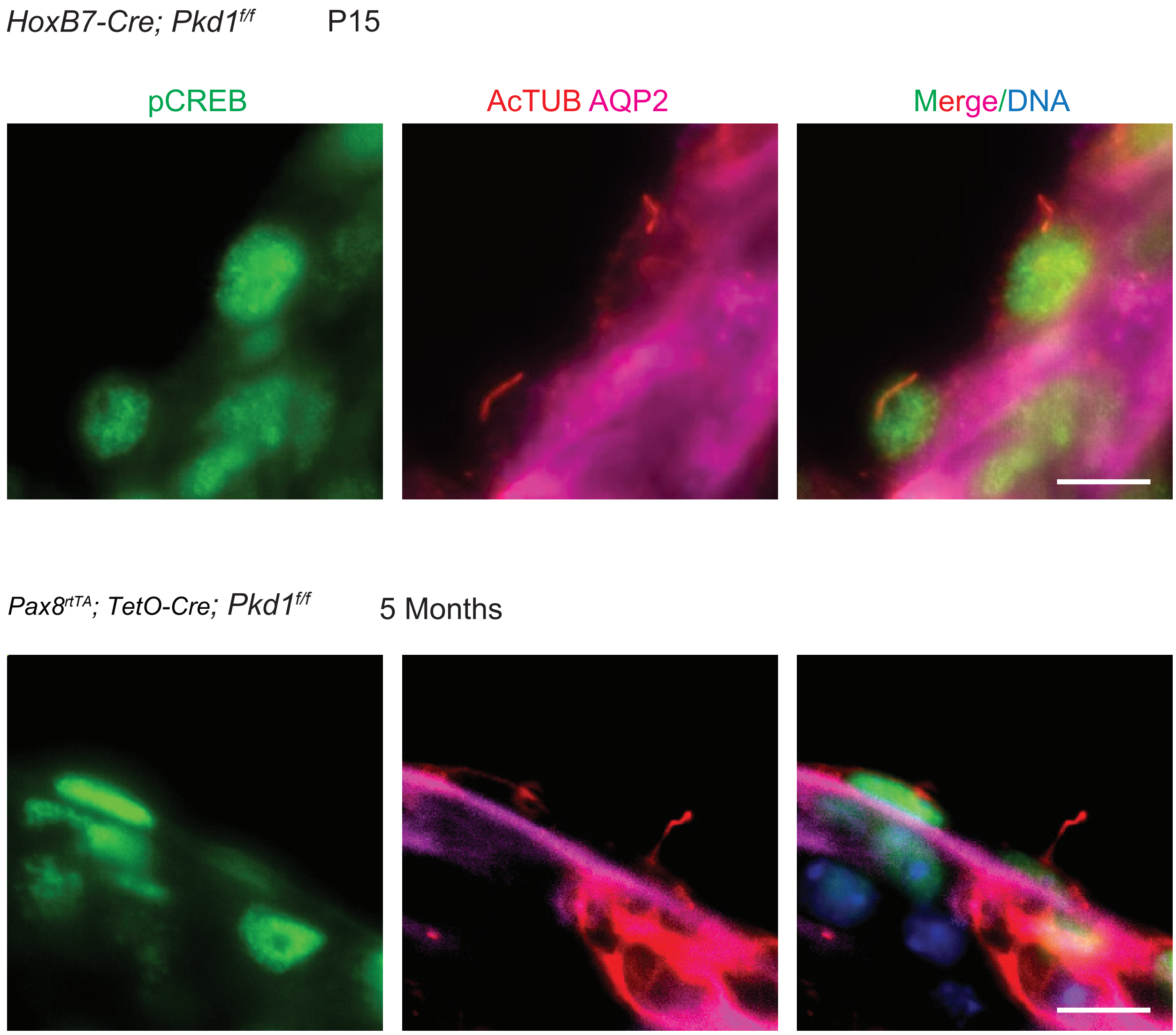
HoxB7-Cre; Pkd1f/f
P15
pCREB
AcTUB AQP2
Merge/DNA
Pax8rtTA; TetO-Cre; Pkd1f/f
5 Months

FIG R2

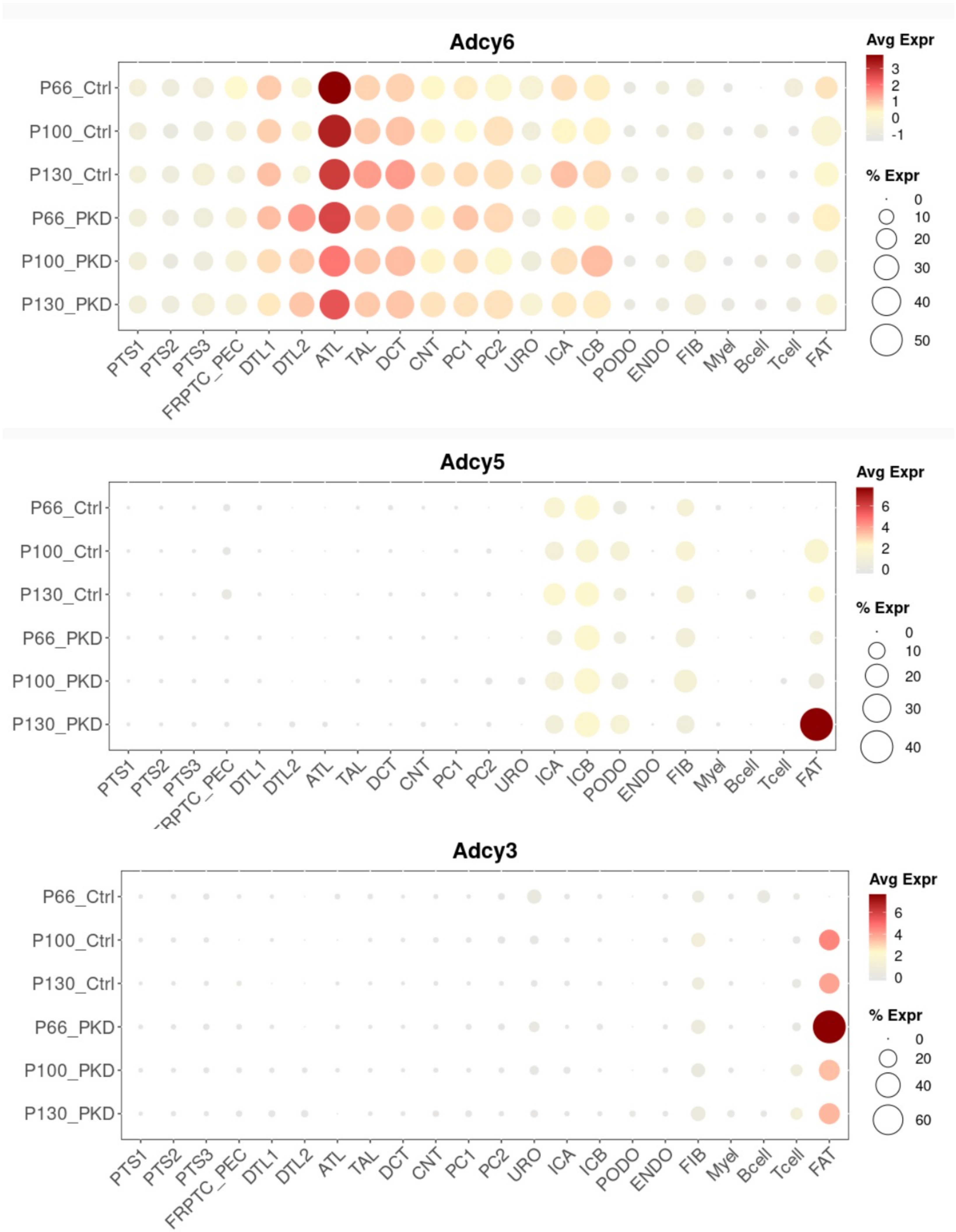
Adcy6
P66_Ctrl
P100_Ctrl
P130_Ctrl
P66_PKD
P100_PKD
P130_PKD
PTS1 PTS2 PTS3 FRPTC_PEC DTL1 DTL2 ATL TAL DCT CNT PC1 PC2 URO ICA ICB PODO ENDO FIB Myel Bcell Tcell FAT
Avg Expr
3 2 1 0 -1
% Expr
0 10 20 30 40 50
Adcy5
P66_Ctrl
P100_Ctrl
P130_Ctrl
P66_PKD
P100_PKD
P130_PKD
PTS1 PTS2 PTS3 FRPTC_PEC DTL1 DTL2 ATL TAL DCT CNT PC1 PC2 URO ICA ICB PODO ENDO FIB Myel Bcell Tcell FAT
Avg Expr
6 4 2 0
% Expr
0 10 20 30 40
Adcy3
P66_Ctrl
P100_Ctrl
P130_Ctrl
P66_PKD
P100_PKD
P130_PKD
PTS1 PTS2 PTS3 FRPTC_PEC DTL1 DTL2 ATL TAL DCT CNT PC1 PC2 URO ICA ICB PODO ENDO FIB Myel Bcell Tcell FAT
Avg Expr
6 4 2 0
% Expr
0 20 40 60

FIG R3

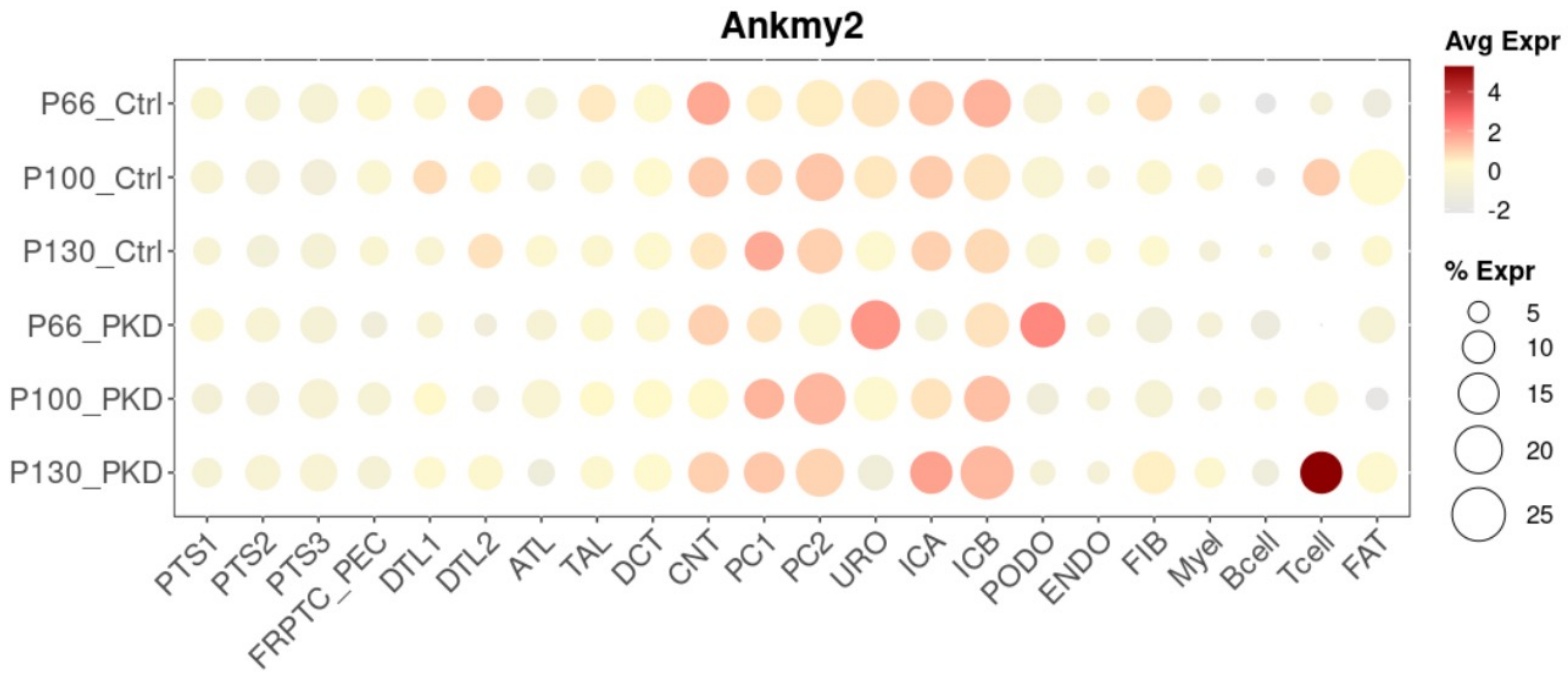

Ankmy2
P66_Ctrl
P100_Ctrl
P130_Ctrl
P66_PKD
P100_PKD
P130_PKD
PTS1
PTS2
PTS3
FRPTC_PEC
DTL1
DTL2
ATL
TAL
DCT
CNT
PC1
PC2
URO
ICA
ICB
PODO
ENDO
FIB
Myel
Bcell
Tcell
FAT
Avg Expr
4
2
0
-2
% Expr
5
10
15
20
25
