# Supplementary material for: Lack of ANKMY2 suppresses kidney cystogenesis in embryonic- and adult-onset polycystic kidney disease
Source: PLoS Genet. 2025 Dec 31;21(12):e1012008. doi: 10.1371/journal.pgen.1012008 (PMC12774363; doi:10.1371/journal.pgen.1012008)
Supplement: Attachment — Submitted filename: ResponseEditorComments_Hwang Rev2 .pdf [file pgen.1012008.s011.pdf]

We wholeheartedly thank the editors for kindly appreciating that we have adequately addressed most of the reviewer concerns in the previous revision. We have now addressed the remaining concerns regarding describing the statistical tests and metrics in the figure legends. These changes have resulted in the following changes:

- Revised figure legends in multiple figures including Figures 1-7 and supplementary figures S1, S3-6.
- Updated Figures 1, 2, 4, 5 and S1 based on the previous comments.
- Updated Figure 8.
- Updated affiliation of Dr. Badgandi.
- Uploaded confirmation from all authors regarding authorship update during revision.

Revised text in the manuscript is marked.

Editorial comments: I have carefully gone through your manuscript and feel that you have adequately addressed most of the reviewer concerns by either new work or by adjusting the text to better describe what was observed. The only concern that I have is that the request to describe the statistical tests in the figure legends was not adequately addressed. The materials and methods list several tests and say that the figures show either mean+/-SD or mean+/-SEM. However. the figure legends lack information about how the data in the figure was analyzed. The figure legends should clearly state the test used to obtain the P values listed and state whether the error bars (if you have them) are SEM or SD.

Thanks for the comments and we are sorry for the oversight.

We have now revised figure legends in multiple figures including Figures 1-7 and supplementary figures S1, S3-6 incorporating these points regarding describing the statistical test used and the error bars. These revised changes are all marked in the manuscript version showing edits.
